# Composite Anion Exchange Membranes Containing a Long-Side Chain Ionomer and Exfoliated Lamellar Double Hydroxides

**DOI:** 10.3390/membranes14120275

**Published:** 2024-12-20

**Authors:** Riccardo Narducci, Suanto Syahputra, Maria Luisa Di Vona, Philippe Knauth, Luca Pasquini

**Affiliations:** 1 LIME Laboratory, Department of Industrial Engineering, University of Rome Tor Vergata, Via del Politecnico 1, 00133 Rome, Italy; divona@uniroma2.it; 2 LIME Laboratory, CNRS, MADIREL (UMR 7246), Campus St Jérôme, Aix Marseille University, 13013 Marseille, France; suanto.syahputra@univ-amu.fr (S.S.); philippe.knauth@univ-amu.fr (P.K.)

**Keywords:** exfoliated LDH, poly(2,6-dimethyl-1,4-phenylene oxide), alkaline stability, ionic conductivity, mechanical and thermal stability

## Abstract

Anion Exchange Membranes (AEMs) are promising materials for electrochemical devices, such as fuel cells and electrolyzers. However, the main drawback of AEMs is their low durability in alkaline operating conditions. A possible solution is the use of composite ionomers containing inorganic fillers stable in a basic environment. In this work, composite anion exchange membranes are prepared from poly (2,6-dimethyl-1,4-phenylene oxide) with quaternary ammonium groups on long-side chains (PPO-LC) and exfoliated Mg/Al lamellar double hydroxide (LDH) as inorganic filler added in different percentages (2, 5, and 10%). The mechanical stiffness of the membranes increases significantly by the addition of exfoliated LDH up to 5%. The ionic conductivity is measured as a function of the temperature in fully humidified conditions and as a function of relative humidity (RH). The maximum conductivity is observed for 5% LDH. The average activation energy for conductivity amounts to 0.20 ± 0.01 eV in fully humidified conditions and >50% RH. Thermogravimetric analysis of membranes before and after alkaline degradation tests (2 M KOH @ 80 °C, 48 h) reveals that the sample with 5% LDH has improved stability (19% vs. 36% of degradation). The stability tests are also investigated, measuring the ionic conductivity and the water uptake. A protective effect of LDH on the alkaline degradation of quaternary ammonium groups is clearly evidenced and opens the way to the use of different compounds and exfoliation methods in the LDH family.

## 1. Introduction

The low alkaline stability of anion exchange membranes (AEMs) is their main drawback, impeding their large-scale use in fuel cells and other electrochemical devices, including water electrolyzers [1,2,3]. The availability of stable ionomers in alkaline conditions would avoid the use of expensive noble metals, like platinum, as electrocatalysts for the oxygen reduction reaction (ORR), reducing the total cost of the overall device [4,5,6].

Various ionomer degradation processes can occur in alkaline media. The cationic groups can degrade following three main pathways: (1) Hoffman elimination (E2), (2) ylide formation, and (3) bimolecular nucleophilic substitution (S_N_2) [7,8,9].

The degradation of the ionomer backbone occurs by the attack of the hydroxide groups on the ether linkage. In the case of polysulfone (PSU), the attack is facilitated by the inductive effect of the sulfone groups [10]. Poly(2,6-dimethyl-1,4-phenylene oxide) (PPO) is expected to have a higher stability because it presents a less activated backbone due to the absence of the sulfone moiety, but the presence of ether links still causes some degradation [11,12,13].

Various strategies have been explored to decrease the degradation in alkaline media [14], including a delocalization of the positive charge, protection by steric hindrance, the introduction of long-side chains, and different architectures of the polymer backbone [15,16,17,18,19,20,21]. The use of composite membranes made by inorganic fillers stable in alkaline environments such as Layered Double Hydroxides (LDHs) has also demonstrated an improvement in mechanical and stability properties [22,23].

LDHs are a family of 2D materials and belong to the group of anionic clays. They are represented by the general formula [M^2+^_1−x_M^3+^_x_ (OH) _2_](A_x/n_^n−^)·mH_2_O, where M^2+^ and M^3+^ can be divalent and trivalent metal ions, x has to be between 0.22 and 0.33, and A^n−^ is the negative counterion that can be easily exchanged [24]. LDH compounds have been synthesized by several methods [25], which include co-precipitation [26], sol-gel synthesis [27,28], and hydrothermal growth [29].

The exfoliation of layered compounds has attracted increasing attention because the resulting unilamellar crystallites or nanosheets have new physical and chemical properties due to their molecular thickness of around 1 nm. Most of the materials found in the literature are “macroanions”, therefore positively charged nanosheets will further broaden the applicability [30,31,32,33,34,35,36]. However, inner surface layers can be inaccessible, which largely restricts their direct applications. The delamination of LDHs into single layers may be the most effective solution to this problem [37]. When exfoliated LDH nanosheets are added to polymers, properties including mechanical performance, thermal stabilization, flame retardation, and even absorption of the resultant nanocomposites can be significantly improved [38].

The exfoliation of LDH leads ideally to single nanosheets (with a thickness of 0.47 nm for MgAl-LDH) and 100–1000 nm lateral size. The swelling of LDH in water is relatively low; for this reason, efficient LDH exfoliation requires suitable solvents and mechanical aids such as ultrasound and mechanical stirring or shaking [39]. Formamide is one of the solvents of choice because it presents a high polarity; LDH layers interact strongly with the carbonyl group of formamide, while the -NH_2_ groups present poor interactions with LDH, anions, and water molecules [40]. Delamination occurs with a fast solvent uptake into a highly swollen phase; the rapid replacement of water by formamide disrupts the original hydrogen-bond network, weakens the interlayer attraction force between the LDH layers, and facilitates progressive exfoliation into single sheets. As reported in the literature, NO_3_^−^-LDH was found to have the best delamination behavior [41].

There are a few examples of the direct use of exfoliated LDH in anion exchange membrane fuel cells (AEMFCs) [42,43], such as ion conductor composite films [44] or hydrogels aligned by external stimuli [45,46]. Chen et al. [47] designed a sandwich structure by enveloping a triple-cation side chain PPO membrane with two layers of quaternized LDH. Ni and Wang [48] developed an in situ growth method for preparing LDH composite AEMs; He et al. prepared a composite membrane with ion-conducting 2D channels based on exfoliated LDH nanosheets and quaternized polyvinyl alcohol via a filtration process [49].

In this study, exfoliated LDH was synthesized and dispersed in poly(2,6-dimethyl-1,4-phenylene oxide) pentyl-trimethylammonium chloride, called in the following PPO-LC (long chain), in different percentages (2, 5, and 10%). The properties of these composites (PPO-LC-LDH), such as ion conductivity, water uptake, swelling, and thermal stability, before and after alkaline degradation tests were investigated and discussed.

## 2. Materials and Methods

### 2.1. Reagents

Poly(2,6-dimethyl-1,4-phenylene oxide) (PPO, MW = 50,000 g/mol), trimethylamine (TMA, 4.2 M in ethanol), n-butyllithium (BuLi, 2.5 M in hexane), tetrahydrofuran (THF, anhydrous), 1,4-dibromobutane, N-methyl-2-pyrrolidone (NMP), formamide, diethyl ether, Mg(NO_3_)_2_·6H_2_O, Al(NO_3_)_3_·9H_2_O, KCl, and NaOH were reagent-grade and purchased from Sigma-Aldrich (St. Louis, MO, USA).

### 2.2. LDH Synthesis

LDH in nitrate form was synthesized by a co-precipitation method with a molar ratio of Mg/Al = 2, corresponding to the formula Mg_0.66_Al_0.33_(OH)_2_(NO_3_)_0.33_. Decarbonated water was used to prepare the two salt solutions and avoid the formation of Mg/Al carbonates. 0.02 mol Mg(NO_3_)_2_·6H_2_O and 0.01 mol Al(NO_3_)_3_·9H_2_O were slowly added in a flask at room temperature under magnetic stirring and a constant nitrogen flux. A solution of 1 M NaOH was added dropwise under magnetic stirring until pH = 10.0. The pH was controlled by in situ potentiometric titration. The resulting slurry was heated for 18 h at 80 °C. The white precipitate was washed with deionized water many times and dried in an oven at 80 °C for 48 h.

### 2.3. LDH Exfoliation

Here, 0.2 g of LDH was dispersed in 100 mL of formamide for 2 days under strong stirring at RT and sonicated for 15 min. The material was stored in a closed Teflon container.

### 2.4. Synthesis of PPO−Precursor

Here, 5 g of dried PPO (41.7 meq) were dissolved in anhydrous THF (500 mL) under nitrogen flux at 55 °C. The solution was cooled to room temperature (RT), and 25 mL of n-BuLi (62.6 meq) was added dropwise until the appearance of a persistent pale yellow coloration. The reaction was carried out for 3 h at 55 °C. The solution was then cooled to −70 °C, and 15 mL of 1,4-dibromobutane (125 meq) were added. A solution was immediately formed, and the reaction was kept under stirring overnight at RT. The product was precipitated in ethanol, and the yellow polymer was filtered, washed repeatedly with fresh ethanol, and dried at 55 °C for 2 days (Degree of functionalization DF = 0.38 by ^1^H-NMR).

### 2.5. Synthesis of PPO-LC

The PPO precursor was dissolved in NMP, and 0.5 g of KI was added to boost the reaction. The TMA was added under nitrogen flux in 5 times excess with respect to the DF because of the well-known volatility of TMA. The solution was kept at 80 °C for 120 h; after this time, the excess of TMA was eliminated by heating the solution at 80 °C for 2 h under vacuum using a liquid nitrogen trap and a rotary pump (2·10^−6^ bar). The polymer was precipitated in diethyl ether, filtered, washed with water, and dried under P_2_O_5_ at RT for 3 days.

### 2.6. Preparation of Composite Membranes

PPO-LC-LDH composite membranes with 2, 5, and 10 wt.% of LDH were prepared by adding the proper amount of filler dispersion in formamide to 20 mL of a 0.05 M solution of PPO-LC in DMSO. The resulting mixture was stirred for 6 h and evaporated to around 10 mL on a heating plate at 100 °C. The viscous solution was then cast onto a Petri dish and heated to 90 °C for 72 h. After cooling to room temperature, the resulting membranes were washed several times in deionized water.

The composite membranes contain initially several anions from the synthesis of PPO-LC and LDH; for this reason, the counterions were systematically exchanged with Cl^−^ or OH^−^ ions before any measurements.

## 3. Characterization Techniques

### 3.1. ^1^H-NMR Spectroscopy

^1^H-NMR spectra were recorded with a Bruker Avance 700 spectrometer (Milan, Italy) operating at 700.18 MHz using deuterated solvents (CDCl_3_, DMSO-d_6_).

### 3.2. X-Ray Powder Diffraction

X-ray powder diffraction (XRPD) patterns were recorded using a Bruker D2 Phaser diffractometer (Milan, Italy) operating at 30 kV and 15 mA, with a step size of 0.02 degrees and a time per step of 1 s, using Cu Kα radiation (1.54 Å) and a multistrip LYNXEYE SSD160 detector (Bruker, Billerica, MA, USA).

### 3.3. Ion Exchange Capacity

The IEC (milliequivalents per gram of dry polymer) was determined by potentiometric acid–base titration. The membranes were treated in 1 M NaOH solution for 2 days to have OH^−^ form and washed in bidistilled water for 2 days to remove the excess base. After drying over P_2_O_5_ for 72 h, the membranes were weighed and immersed in a 0.02 M HCl solution. The acid solution was then back-titrated with a 0.02 M NaOH solution.

### 3.4. Thermogravimetric Analysis (TGA)

The high-resolution TGA analysis was performed using a TGA Q500 (TA Instruments, New Castle, DE, USA). Around 8 mg of Cl^−^ form membrane was placed in Pt sample holders and heated with a maximum rate of 3 K/min from 30 to 800 °C in air. Comparative thermograms were recorded after the degradation of the ionomers in 2 M KOH at 80 °C for 3 days.

### 3.5. Water Uptake

Membrane samples dried over P_2_O_5_ for 3 days were weighed (m_dry_) and then immersed for 24 h in liquid water in a closed Teflon vessel at 25 °C. The excess water was carefully wiped off and the membrane mass was determined (m_wet_). The water uptake WU can be calculated according to Equation (1):

WU = [(m_wet_ − m_dry_)/m_dry_] × 100 (1)

The uncertainty in the measurements was ~±5%.

### 3.6. Membrane Density and Volume Swelling

A membrane sample (~3 cm × 2 cm) was first dried for one day over P_2_O_5_ at room temperature, and then the weight and the a, b, and c dimensions were accurately determined by a micrometer. The dried membrane was immersed in deionized water at 25 °C and equilibrated for 24 h, then the weight and a, b, and c dimensions were again determined. The volume swelling (Sv) and the dry density (D_dry_) were calculated as follows:

Sv = 100 × (V − V_0_)/V_0_ (2)

D_dry_ = m_dry_/V_0_ (3)

where V_0_ and V were the volumes of the dry and swollen membrane calculated from its length, width, and thickness. The uncertainty in the measurements was ~±5%.

### 3.7. Stress–Strain Tests

The mechanical properties of composite membranes were investigated using an ADAMEL Lhomargy DY30 (Saint Baldoph, France) traction machine at 25 °C and ambient humidity at a constant crosshead speed of 5 mm/min on 5 × 0.5 cm rectangular samples. Particular attention was given to the macroscopic homogeneity of the membranes, and only homogeneous membranes were used for the mechanical tests.

### 3.8. Ionic Conductivity

All the analyses were performed using a VSP-300 potentiostat (BioLogic Science Instruments, Seyssinet-Pariset, France).

Before the measurements, the samples in Cl^−^ form were immersed in deionized water for 24 h at 25 °C, and the excess water on the surface was removed with absorbing paper just before the introduction in the conductivity cell. For the through-plane conductivity measurements, the membranes were introduced inside a hermetically closed Swagelok cell with stainless steel electrodes to guarantee full humidification. The impedance measurements were performed between 1 Hz and 6 MHz with a signal amplitude of 20 mV. The resistance of the membranes in fully humidified form was obtained at 25, 45, 60, and 80 °C from a Nyquist plot by non-linear least-square fitting. The equivalent circuit was a classical series arrangement of a resistance R in series with a parallel circuit of resistance and a constant phase element. The ionic conductivity σ was calculated using Equation (4) with the resistance R, the membrane thickness d (typically 50–60 μm), and the electrode area A = 0.264 cm^2^.

σ = d/(A·R)(4)

For the in-plane conductivity measurements, the membranes, prepared as already described and cut in a rectangular shape with 5 cm length and 0.5 cm width, were mounted in a four-probe BekkTech conductivity cell BT-112 (Scribner, Southern Pines, NC, USA). The measurements were realized by varying the temperature from 25 to 80 °C and the relative humidity from 30 to 95% in a climatic chamber (FDM model 40C180V25, FDM—Environment Makers, Rome, Italy). Impedance spectroscopy (a.c. amplitude: 20 mV, frequency range: 1 Hz–6 MHz) and linear sweep voltammetry (voltage between −0.02 and 0.02 V, rate 1 mV/s) were applied using the already mentioned potentiostat.

### 3.9. Accelerated Alkaline Degradation Tests

The degradation tests were carried out in 2 M KOH at 80 °C for 3 days. After the treatment, the membranes were rinsed several times in deionized water and carefully dried on paper, after which they were stored in a closed Teflon container.

## 4. Results and Discussion

**Figure 1:**
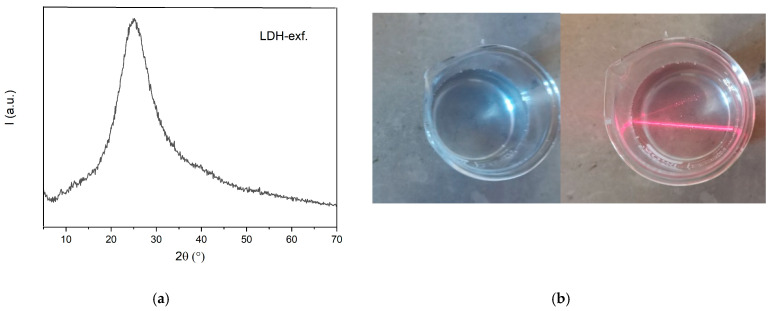
(**a**) XRPD diffractogram of exfoliated LDH; (**b**) photograph of a colloidal suspension of exfoliated LDH nanosheets. The red light beam was incident from the side to demonstrate the Tyndall effect.

The XRPD pattern of exfoliated LDH is shown in Figure 1. The sharp basal reflections of the LDH were absent, indicating that the layer structure has collapsed; the peak at 25° is related to the scattering of formamide as reported in the literature [37,50], showing the effectiveness of the delamination.

A clear Tyndall light scattering is observed in Figure 1b. The resulting colloidal suspension is very stable, and no sediment is observed upon long-term standing over 1 year.

**Figure 2:**
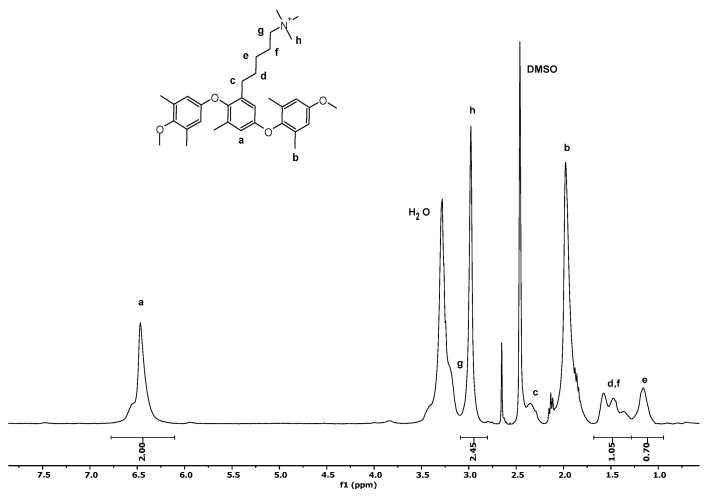
^1^H-NMR spectrum of PPO-LC with DA = 0.27 in DMSO-d_6_.

Figure 2 shows the ^1^H-NMR spectrum of PPO−(CH_2_)_5_−TMA (PPO-LC). The degree of amination (DA) of PPO-LC is obtained from the integration of the aromatic hydrogens of PPO (a, 2H) and the hydrogens of methyl groups on the ammonium ions (h, 9H), resulting in DA = 0.27.

Table 1 shows the water uptake, volume swelling (S_V_), density in anhydrous conditions (D_dry_), and IEC values of PPO-LC-LDH membranes at different wt.% of filler and with different counterions. As expected, an increase in the percentage of filler in the membrane (Table 1a,b) decreased both the WU and Sv and increased the density; these observations can be attributed to an enhanced interaction of the LDH nanosheets with the polymer leading to a greater compactness of the macromolecular chains in the composites. With OH^−^ as a counterion (Table 1b), the results present the same trend; the absolute values of WU are higher due to the greater hydration of OH^−^ with respect to Cl^−^. The IEC decreased by increasing the amount of LDH. After alkaline degradation (Table 1c), surprisingly low WU and Sv values are observed for the pristine sample, probably related to the large loss of quaternary ammonium groups. The highest hydration is indeed observed for the 5% sample, which corresponds to the best protection against loss of quaternary ammonium groups, as evidenced below.

**Figure 3:**
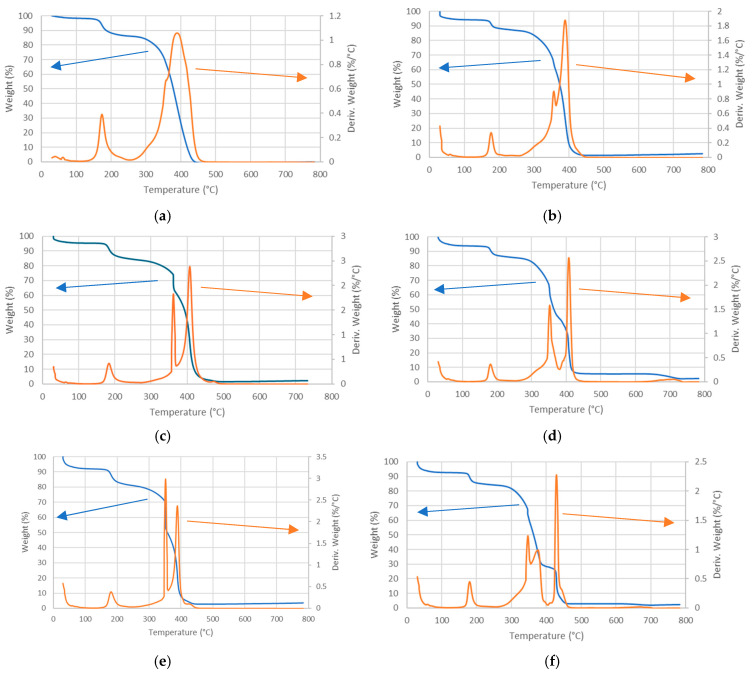
High-resolution thermogravimetric analysis in the air of PPO-LC-LDH membranes before and after accelerated alkaline degradation test: mass loss in blue and derivative thermograms in orange. (**a**) PPO-LC 0% LDH, (**b**) after degradation; (**c**) PPO-LC 2% LDH, (**d**) after degradation; (**e**) PPO-LC 5% LDH, (**f**) after degradation.

Figure 3a–f shows the high-resolution thermogravimetric analysis of various PPO-LC with 0 to 5% LDH membranes before and after accelerated alkaline degradation. The peaks in the derivative thermograms (orange curves) indicate the principal mass loss phenomena.

In pristine PPO-LC (Figure 3a), water evaporation is noticeable below 100 °C. The first peak around 180 °C can be attributed to the loss of TMA, in agreement with a similar ionomer containing trimethylammonium groups [51]. By integrating the peak area (corresponding to the amount of TMA groups) between 160 and 230 °C, one observes that about 12% of the total dry mass (Table 2) is lost in the non-degraded membrane in good agreement with the degree of amination of 0.26 (corresponding to 11%). This peak is significantly reduced after the alkaline degradation treatment (Figure 3b). The mass loss, integrated over the same temperature range, of about 8% corresponds to a decrease in the ammonium groups by about 36% (Table 2).

The second broad peak starting around 300 °C has a maximum at about 390 °C and a shoulder at 355 °C. It can be attributed to the decomposition of partially oxidized and cross-linked PPO, as shown previously [52,53]. The shoulder at 355 °C is the trace of the decomposition of methyl and pentyl groups, which decompose prior to the main chain. Considering the degree of functionalization of PPO-LC, the mass loss should be about 25%, in good agreement with the experiment. The PPO backbone decays at around 390 °C. The remaining mass above 360 °C (60%) is in good agreement with the calculated value for the residual polymer backbone. There is no mass residue above 450 °C.

After the alkaline degradation treatment (Figure 3b), the broad peak with a shoulder transforms into a sharp double peak with identical maximum temperature. Similar observations were reported previously for quaternized PPO [10]. There is a small residual mass above 500 °C that might be attributed to potassium oxide, due to an incomplete rinsing of the membrane after the alkaline degradation. The peak with a maximum near 355 °C, attributed to the methyl and pentyl groups, is reduced after the alkaline treatment. Indeed, the thermal decomposition of PPO starts with the cleavage of the ether linkages and the simultaneous removal of the methyl and pentyl groups [53]. A similar mechanism is certainly observed during the alkaline degradation.

For samples containing LDH (Figure 3c–f), the thermograms are quite similar in terms of degradation temperatures. At higher temperatures, the decomposition reactions of the polymer overlap with those of LDH, reported above 280 °C and up to 500 °C, including the loss of structural water, dihydroxylation of the brucite-like layer, and removal of interlayer chloride ions, giving a mixture of oxides. However, the small amount of LDH does not allow us to observe them clearly. Nevertheless, significant changes in mass loss can be noticed in samples containing LDH. Below 100 °C, the observed water loss is higher, evidently due to the presence of hydrophilic LDH. The thermolysis of TMA occurs around 180 °C. The amount of TMA groups degraded by alkaline treatment, calculated again from the peak area, is only 19% in the 5% LDH sample (Table 2), which shows a protective effect of LDH against the alkaline degradation of TMA groups. In the 5% sample, the degradation of chloromethylated chains (350 °C) and backbone (395 °C) starts at slightly lower temperatures. The peak of the PPO main chain degradation is shifted to slightly higher temperatures (maximum at 410 °C), probably due to the interaction with the inorganic component. The mass loss belonging to the main chain is clearly lower after the alkaline treatment, due to cleavage of ether links; some smaller chains probably degrade at lower temperatures (shoulder or small peak at 380 °C in Figure 3d,f, respectively). There is a small residual mass at 700 °C, belonging to oxides due to the decomposition of the LDH [54], which is consistent with the respective amount of LDH. Furthermore, in both LDH-containing degraded membranes, there is a small mass loss starting around 650 °C, which might result from a loss of CO_2_ due to a small amount of potassium carbonate after treatment in an alkaline solution.

**Figure 4:**
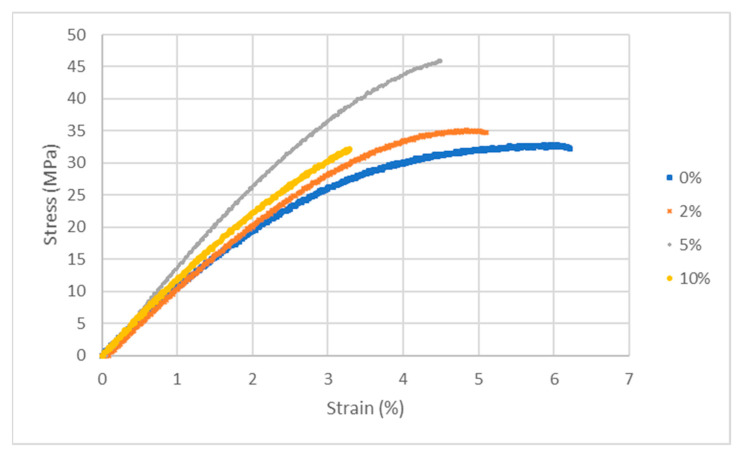
Stress–strain curves of PPO-LC with various % content of LDH.

A summary of the mechanical properties of the various membranes is reported in Figure 4 and Table 3. The mechanical properties improve when adding 2% LDH and reach the maximum at 5%, but degrade for the 10% sample. The increase in Young’s modulus, yield stress, and ultimate stress show a stiffening of the ionomer by the addition of LDH filler, which is consistent with a slight reduction of the elongation at break. The highest amount of inorganic filler leads probably to the formation of aggregates and therefore worsens the contact between the organic and inorganic parts, reducing the mechanical properties. The alkaline-degraded membranes become very brittle at ambient humidity, which does not allow for conducting mechanical tests.

The Cl^−^ ion conductivity of various membranes in fully humidified conditions is reported as a function of temperature in Table 4. There is no significant difference between conductivities measured through-plane and in-plane and with increasing and decreasing temperature (cool). Furthermore, data measured after a 3-day accelerated degradation test in 2 M KOH at 80 °C are reported. The as-cast ionic conductivity increases with the addition of LDH, with a maximum of 5% LDH. Further addition leads to a decrease in conductivity. The conductivity after accelerated degradation tests is also highest for PPO-LC 5% LDH.

The Cl^−^ ion conductivities of the various PPO-LC membranes as a function of temperature and relative humidity (RH) are summarized in Table 5. One can notice that the reference PPO-LC membrane without LDH addition reaches a conductivity above 1 mS/cm only at 95% RH. Furthermore, the conductivity decreases above 60 °C and 50% RH, showing an instability of the membrane. Instead, conductivities above 1 mS/cm are already measured at 70% RH with 2% LDH (at 80 °C) and 5% LDH addition (above 45 °C). Furthermore, the ionic conductivity becomes unstable only at 80 °C and 95% RH. Both results clearly show the superior performance of membranes with LDH addition. The best properties are again observed for membranes with 5% LDH. The stabilization of PPO-LC by LDH is thus clearly evidenced.

**Figure 5:**
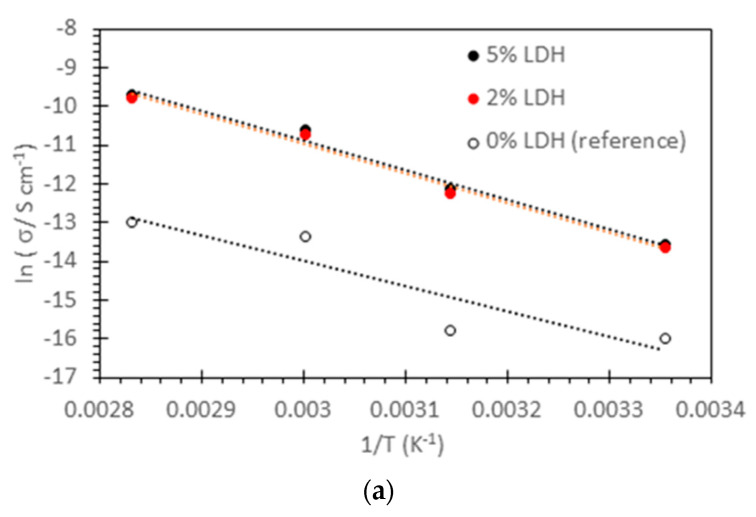

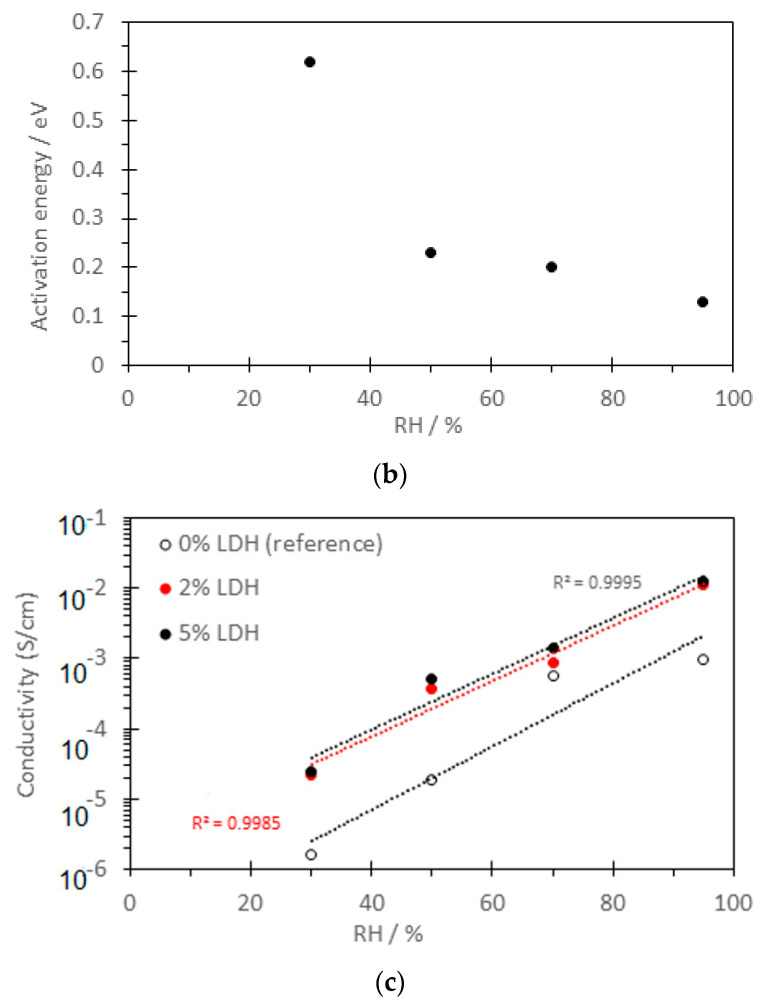
(**a**) Temperature dependence of the Cl^−^ ion conductivity of membranes at 30% RH, (**b**) average activation energies as a function of RH, and (**c**) RH dependence of membrane conductivity.

Figure 5a shows the temperature dependence of the Cl^−^ ion conductivity in Arrhenius representation at 30% RH. The activation energy at 30% RH is quite similar for the 3 membranes, with an average value of (0.62 ± 0.04) eV. Significantly lower activation energies, in the order of 0.2 eV, are observed for higher relative humidities, indicating a change in the conduction mechanism (Figure 5b). At very low humidity, contact ion pairs are probably formed with a “hopping” transport mechanism of chloride ions, whereas water-assisted migration is expected at high humidity. The RH dependence of conductivity can be described by exponential relations, as shown in Figure 5c.

## 5. Conclusions

In this work, we studied the effect of up to 10 percent of exfoliated MgAl-LDH on the properties of composite anion exchange membranes with PPO backbone and quaternary ammonium groups linked by a long alkyl chain. The maximum mechanical stiffness and Cl^−^ ion conductivity are observed at 5% of filler. The beneficial effect of the exfoliated LDH is highlighted by accelerated alkaline degradation tests in 2 M KOH at 80 °C for 3 days, where the protection against the loss of quaternary ammonium groups is evidenced by thermogravimetric analysis. From these results and the inexpensive synthesis of the exfoliated materials, these composite membranes appear promising for use in AEMFCs. Future research will be directed towards the use of ionomers with higher IEC and larger and better structured LDH platelets obtained by a different synthesis, such as the urea method.

## Figures and Tables

**Table 1:** (**a**–**c**) Membrane properties of PPO-LC-LDH: water uptake (WU), swelling (Sv), density in anhydrous conditions (D_dry_), and IEC values.

(a) PPO-LC-LDH (Cl^−^)				
% LDH	WU (%)	S_V_ (%)		D_dry_ (g/cm^3^)
0	32.0	-		-
2	30.3	39.6		1.07
5	21.3	21.3		1.24
10	14.6	15.9		1.27
**(b) PPO-LC-LDH (OH^−^)**				
% LDH	WU (%)	Sv (%)	D_dry_ (g/cm^3^)	IEC (meq/g)
0	46	-	0.90	-
2	41.2	46.0	1.09	1.27
5	39.8	42.0	1.23	1.24
10	34.3	29.0	1.35	1.20
**(c) PPO-LC-LDH (OH^−^) After Accelerated Degradation Test (2 M KOH, 80 °C, 3 Days)**				
% LDH	WU (%)	S_v_ (%)		D_dry_ (g/cm^3^)
0	22.0	14.6		-
2	25.8	24.1		1.09
5	39.8	36.6		1.22
10	33.7	25.7		1.24

**Table 2:** Thermogravimetric analysis of the alkaline degradation of quaternary ammonium groups.

% LDH	Mass Loss Around 180 °C (%)	Variation (%)
0	12.2	36
After Degradation	7.8	
2	12.0	28
After Degradation	8.7	
5	11.2	19
After Degradation	9.1	

**Table 3:** Mechanical properties of PPO-LC-LDH membranes from tensile stress–strain tests.

% LDH	Young’s Modulus (MPa)	Yield Stress (MPa)	Ultimate Stress (MPa)	Elongation @ Break (%)
0	1020 ± 20	20 ± 6	32 ± 2	6 ± 1
2	1040 ± 25	15 ± 1	32 ± 4	4 ± 1
5	1380 ± 2	42 ± 1	44 ± 3	4 ± 1
10	1150 ± 7	7 ± 1	31 ± 2	3 ± 1

**Table 4:** Cl^−^ ion conductivity in fully humidified conditions as a function of temperature of various membranes as cast and after degradation during 3 days in 2 M KOH at 80 °C.

	PPO-LC (mS/cm)		PPO-LC 2%LDH (mS/cm)		PPO-LC 5%LDH (mS/cm)		PPO-LC 10%LDH (mS/cm)
Temperature	As Cast	After Degradation	As Cast	After Degradation	As Cast	After Degradation	As Cast
25 °C	1.0	0.6	2.6	0.9	1.6	1.3	2.6
45 °C	1.5	0.9	4.2	1.3	3.7	2.1	4.0
60 °C	2.0	1.3	5.4	1.7	6	2.5	5.1
80 °C	2.4	2	7.3	2.3	9.9	3.9	6.8
60 °C cool	1.9	1.3	5.1	2	8.1	3.1	5.0
45 °C cool	1.5	1.2	3.9	1.5	6.3	2.6	3.7
25 °C cool	0.9	0.9	2.5	0.9	4	1.5	2.4

**Table 5:** Cl^−^ ion conductivity (S/cm) of PPO-LC membranes as a function of temperature and relative humidity.

Membrane	T/°C	RH/%			
		30	50	70	95
PPO-LC 0% LDH	25	1.1 10^−7^	2.4 10^−6^	1.7 10^−5^	1.4 10^−3^
	45	1.4 10^−7^	1.0 10^−5^	3.7 10^−5^	1.6 10^−3^
	60	1.6 10^−6^	1.9 10^−5^	5.7 10^−4^	9.8 10^−4^
	80	2.3 10^−6^	1.3 10^−5^	9.7 10^−5^	3.3 10^−4^
PPO-LC 2% LDH	25	1.2 10^−6^	1.5 10^−4^	4.3 10^−4^	7.3 10^−3^
	45	4.8 10^−6^	2.2 10^−4^	9.8 10^−4^	8.5 10^−3^
	60	2.2 10^−5^	3.8 10^−4^	8.6 10^−4^	1.2 10^−2^
	80	5.6 10^−5^	5.9 10^−4^	1.8 10^−3^	4.3 10^−3^
PPO-LC 5% LDH	25	1.3 10^−6^	1.9 10^−4^	8.0 10^−4^	3.9 10^−3^
	45	5.3 10^−6^	5.9 10^−4^	1.6 10^−3^	8.8 10^−3^
	60	2.5 10^−5^	5.1 10^−4^	1.4 10^−3^	1.3 10^−2^
	80	6.2 10^−5^	5.8 10^−4^	2.7 10^−3^	8.4 10^−3^

## Data Availability

The raw data supporting the conclusions of this article will be made available by the authors on request.
